# Items

**Published:** 1881-04

**Authors:** 


### Items.

RUSH MEDICAL COLLEGE.—The Thirty eighth Annual Commencement of Rush College occurred on Tuesday afternoon, February 22, at Central Music Hall. This event marked the close of the most prosperous year of the life of the college. The number of the graduates receiving the ordinary degree was 170. This is, we believe, the largest class ever graduated by any college west of Philadelphia. In addition to the ordinary degrees, the honorary degree of Doctor of Medicine was conferred upon Dr. W. W. Allport, the well known dentist of Chicago, in consideration of his labors for the elevation of the profession of dentistry to a specialty of medicine; and upon Dr. S. D. Jacobson, President of the Medical Board of Cook County Hospital, as a testimonial to his worth as a clinical teacher and as a medical gentleman.

The exercises of the Commencement were lengthy and interesting. The audience filled the Music Hall to overflowing. Prayer was offered by Bishop Cheney. The Secretary of the college, Prof. Etheridge, read his report of the work of the college year. The statistical information of the report was as follows: There had been "16 special students, 170 graduates and 319 undergraduate students—total, 505." Of the class graduated, 84 per centum held either literary degrees or teacher's certificates from County Superintendents of Schools. Of the class, 33 had attended the college three years, and 146 had taken two courses of lectures in the college. Five years ago, the class contained only 3 three-years men, and only 36 had spent their two years in this institution.

The degrees were formally conferred by President Allen. A

brief and sensible "Valedictory for the Class" was delivered by Dr. C. T. Clark, after which a very eloquent and impressive address to the graduates was delivered by Prof. DeLaskie Miller.

The doctorate address was then delivered by President Allen. It considered the history and progress of Rush Medical College from its foundation to the present time. It compared this school with others in this country and abroad, and was, in general, a discussion of the question of medical colleges and medical college progress in many of its bearings and aspects.

The occasion was signalized by the President appearing in black gown and Oxford cap.

After the Commencement exercises were over, the Alumni Association held a business meeting. The following officers were elected for the ensuing year: President, Dr. J. L. Whitley, of Osage, Iowa; First Vice President, Dr. J. B. Hamilton, of Washington, D. C.; Second Vice President, Dr. Philip Leach, Class of 1881; Secretary and Treasurer, Dr. F. A. Emmons; Executive Committee, Drs. J. E. Owens, W. T. Belfield and Otto T. Freer.

In the evening the Faculty of the college entertained the Alumni and invited guests at a banquet at the Tremont House.

The toasts were as follows:

1. *Clinical Medicine*, Prof. D. T. Nelson.

"Laying his hand upon many a heart,
Had healed it forever."

2. *Medicine and Dentistry*, Dr. W. W. Allport.

"Sure the shovel and tongs
To each other belongs."

3. *The Sisters of Medicine*, Hon. Geo. Gardner.

"And loved to plead, lament and sue."

4. *The Profession of Medicine*, Dr. Norman Bridge.

" * * * but when ill indeed,
E'en dismissing the doctor don't always succeed."

5. *The First Dose of Medicine*, Dr. C. S. Shepard, Of the Class of 1881.

"And so he was reduced at last,
To practice in a country town."

6. *Medicine and Civil Service Reform*, Dr. F. C. Henrotin.

"At this the Mayor and Corporation
Quaked with a mighty consternation."

7. *Medicine in Illinois*, Dr. Geo. Wheeler Jones,

Of Danville, Ill.

"Who comes and asks you how's your liver,
And where you ache, and whether you shiver."

8. *Alma Mater*, Prof. Henry M. Lyman.

"And gladly wolde she lerne, and gladly teche."

*Address of the President of the Alumni Association*,
Dr. Solon Marks, of Wisconsin.

AN APPEAL TO THE MEDICAL PROFESSION.—Although the *Index Medicus*, during its second year, has not been so great a loss financially as in its first, its actual expenses are not yet fully covered by subscription, the mass of material and the labor involved making it a far more costly publication than any similar periodical issue. The value of the work has, however, been so thoroughly recognized by those who have practically tested it that a number of American subscribers have volunteered, in addition to their subscription, to contribute to a guarantee fund, for the purpose of securing the publisher against further loss, and, at the same time, to give the medical profession another opportunity to place the publication upon a self-supporting basis. Thus it has been decided to give it another year's trial, and it is hoped that the example set by an appreciative and generous minority will not prove fruitless. Only 200 more subscriptions are required to permanently establish a publication which is of incalculable benefit to the whole medical world. Even where individual subscriptions cannot be afforded. some personal influence among the medical societies and libraries, a few words of earnest commendation to medical friends, or through the medical press, would accomplish the desired end.

It is almost unnecessary to say that the suspension of the *Index* would prove an irreparable loss to the entire profession. It is doubtful whether the publication could ever be revived under similar favorable conditions, viz.:

The vast material and admirable system at the National Medical Library, in Washington.

The capacity of the editors, equaled only by their personal sacrifice and devotion to their laborious task.

A publisher who, after all his discouragement and loss, again offers his services without expecting any remuneration until the enterprise is established.

It would be presumptuous on the part of the publisher to dwell on the merits of the work itself when the most prominent men of the profession have given it their hearty support and unqualified commendation. But he should be permitted, on this very ground, to ask every one interested in the promotion of medical science and literature to aid the undertaking, either in the way of individual subscription or personal influence.

The *Index Medicus* is a monthly classified record of the current medical literature of the world, compiled under the supervision of Dr. John S. Billings, Surgeon, U. S. A., and Dr. Robert Fletcher, M.R.C.S., England. It records the titles of all new publications in medicine, surgery and the collateral branches received during the preceding month. These are classed under subject-headings, and followed by the titles of valuable original articles upon the same subject, found, during the like period, in medical journals and transactions of medical societies. The periodicals thus indexed comprise all current medical journals and transactions of value, so far as they can be obtained.

The *Index Medicus* is published monthly, and supplements all the leading medical journals (American and foreign) as a current guide and general index to all.

Subscription price per annum, postage prepaid, for United States and Canada, $6. *Sample copies sent free on application.* The addresses of probable subscribers are desired from the friends of the enterprise. Address F. Leypoldt, publisher, 13 and 15 Park Row, New York.

Subscriptions abroad will be received by Trübner & Co., 57 Ludgate Hill, London; J. H. Baillière et Fils, Rue Hautefeuille, 19, Paris; K. F. Köhler, Poststrasse, 16, Leipzig.

AMERICAN MEDICAL ASSOCIATION.—The thirty-second annual session will be held in Richmond, Va., on Tuesday, Wednesday, Thursday, and Friday, May 3, 4, 5, 6, 1881, commencing on Tuesday at 11 A. M.

" The delegates shall receive their appointment from such permanently organized State Medical Societies as are recognized by *representation in their respective State Societies*, and from the Medical Department of the Army and Navy, and the Marine Hospital Service of the United States."

"Each State, County, and District Medical Society entitled to representation shall have the privilege of sending to the Association one delegate for every ten of its regular resident members, and one for every additional fraction of more than half that number: *Provided*, however, that the number of delegates for any particular State, territory, county, city, or town shall not exceed the ratio of one in ten of the resident physicians who may have signed the Code of Ethics of the Association."

Secretaries of Medical Societies as above designated are earnestly requested to forward, *at once*, lists of their delegates.

SECTIONS.—"The Chairmen of the several Sections shall prepare and read in the general sessions of the Association, papers on the advances and discoveries of the past year in the branches of science included in their respective Sections." * * * * —By-Laws, Art. II., Sec. 4.

*Practice of Medicine, Materia Medica and Physiology.*—Dr. Wm. Pepper, 1811 Spruce st., Philadelphia, Pa., Chairman; Dr. T. A. Ashby, Baltimore, Md., Secretary.

*Obstetrics and Diseases of Women and Children.*—Dr. Jas. R. Chadwick, cor. Marlborough and Clarendon sts., Boston, Mass., Chairman; Dr. Joseph Taber Johnson, Washington, D. C., Secretary.

*Surgery and Anatomy.*—Dr. Hunter McGuire, Richmond, Chairman; Dr. Duncan Eve, Nashville, Tenn., Secretary.

*State Medicine.*—Dr. Jas. T. Reeve, Appleton, Wis., Chairman; Dr. R. G. Jennings, Little Rock, Ark., Secretary.

*Ophthalmology, Otology and Laryngology.*—Dr. Dudley S. Reynolds, Louisville, Ky., Chairman; Dr. Swan M. Burnett, Washington, D. C., Secretary.

*Diseases of Children.*—Dr. A. Jacobi, 101 W. 34th st., New York, Chairman; Dr. T. M. Rotch, 77 Marlborough st., Boston, Mass., Secretary.

A member desiring to read a paper before any Section should forward the paper, or its *title*, and *length* (not to exceed twenty minutes in reading), to the Chairman of the Committee of Arrangements at least one month before the meeting.—*By-Laws.*

*Committee of Arrangements.*—Dr. F. D. Cunningham, Richmond, Va., Chairman.

*Amendment to the By-Laws* offered by Dr. J. M. Keller, Ark.—In the election of Officers and the appointment of committees by this Association and its President, they shall be confined to members and delegates present at the meeting, except in the Committee of Arrangements, Climatology, and Credentials.

W. B. ATKINSON,
*Permanent Secretary.*

AN INTERESTING MORTALITY EXHIBIT.—The Health Department has compiled the following details from the record of deaths by phthisis pulmonalis in the city of Chicago for the year ending December, 1880. It is said to be the most complete abstract ever made up in the department:

Deaths by months: January, 67; February, 65; March, 93; April, 74; May, 60; June, 67; July, 66; August, 64; September, 61; October, 75; November, 56; December, 52—total, 800.

Occupations: Males—Actors, 3; artists, 1; bakers and confectioners, 2; barbers, 3; bell-maker, 1; blacksmiths, 10; book-binders, 3; boot and shoe makers, 9; brewers, 3; brass-finishers, 3; butchers, 6; cigar makers, 11; clerks and salesmen, 30; coopers, 5; cooks, 2; engineers and firemen, 5; farmers and gardeners 9; grocers, 5; gunsmiths, 2; hatters, 4; lawyers, 6; laborers, 75; lithographers, 2; machinists, 13; masons and bricklayers, 6; merchants, 20; moulders, 4; musicians, 2; peddlers, 2; packers, 2; painters, 10; physicians, 4; photographers, 1; plasterers 2; plumbers, 3; police, 1; painters, 17; porters, 5; priests, 2; sailors, 3; saloon-keepers, 9; stonecutters, 1; tailors, 20; tanners, 3; preachers, 4; tinsmiths, 5; telegraphers, 2; teamsters, 3; undertakers, 3; waiters, 8; woodworkers, 35; book-keepers, 15—total, 400.

Females—Dressmakers, 7; laundresses, 7; milliners, 2; seamstresses, 25; housewives, 110; tailoresses, 5; servants, 10; unknown, 35; under 15 years, 200—total, 400.

TREATMENT OF MEMBRANOUS SORE THROAT.—At a recent meeting of the Academy of Medicine of Paris, Dr. Viard made a communication on this subject. He considers that membranous sore throat is primarily a local affection, which does not become general for five or six days; during the first period the diphtheria may be cured by cauterization (*Medical Press and Circular*). During the last eighteen months he has had twenty-six cures out of twenty-eight cases. Wrapping his finger in a rough cloth he removes the false membrane, leaving in its place a bleeding surface; then he cauterizes with nitrate of silver; four or five such cauterizations neutralize the diphtheritic poison. General tonic treatment is employed at the same time.

PILOCARPINE: ITS GENERAL EFFECTS AND ITS ACTION IN SYPHILIS.—Dr. Lewin, of La Charité Hospital, Berlin, has been experimenting on the action of pilocarpine on the salivary and sudoriparous glands. In the course of three years and a half he has treated thirty-two patients affected with different forms of syphilide by subcutaneous injection of pilocarpine. Seventy-eight per cent. of the patients were cured. Of seven cases two were of serious form, and had resisted energetic mercurial treatment; the cure was incomplete, and it was necessary to have recourse to injections of corrosive sublimate to complete it. In five other cases the treatment had to be suspended on account of inter-current complications (endocarditis, hæmoptysis, collapse).

The patients who were cured by the aid of pilocarpine showed large condylomata, various exanthemata, pharyngeal lesions, one a gummatous periostitis, and one ulcer of the leg.

The mean duration of the treatment was eighty-four days. The dose injected each time was usually 15 milligrames. The cure would be shorter if patients would have daily injections; but as soon as amendment of the symptoms begins they require less and less frequent applications of the remedy.

Pilocarpine seems to prevent relapses with greater surety than mercury or vegetable depuratives. But in respect to facility of application, certainty of result, and rapidity of cure, this medication is inferior to injections of corrosive sublimate, and often leaves behind it extreme sensibility to the influences of temperature, which obliges patients, after the cure, to keep their room for some time for fear of arthritic and rheumatic troubles.

According to the experience of Lewin and others, pilocarpine and its salts act especially on the salivary and sudoriparous glands. The symptoms which its use may cause, and which may oblige us to suspend the treatment are, nausea, vomiting, cephalalgia, cramps, trembling of the hands, swelling of the submaxillary glands, weakness, loss of sleep, erysipelas of the face, and stomatitis.

HYDRORRHŒA GRAVIDARUM.—M. Stapfer, in a *Thése de Paris*, has laboriously collected the materials that exist for arriving at some knowledge of this obscure subject (*Med. Times and Gazette*). The multitude of synonyms under which the symptoms have been described—dropsy of the womb, dropsy of the membranes, premature and spontaneous rupture of the membranes, false waters, metrorrhœa—indicate the different opinions that have been held. The name hydrorrhœa, M. Stapfer thinks, has been given to watery discharges resulting either from premature rupture of the membranes, from transudation of amniotic fluid, from rupture of a supernumerary ovum, from rupture of a cyst, from exudation from the uterine wall, from the decidua, the chorion, the amnion, the vessels or glands of the neck and body of the uterus. In the presence of such confusion, he thinks it impossible yet to frame a definition of the disease. From the evidence before him he comes to the following conclusions: The hydrorrhœa which appears in the early months of pregnancy, and which is the exception, seems only explicable by supposing the existence of a "*hydropérione*" between the decidua vera and decidua reflexa. That which comes on in the latter months results from a collection of fluid outside, or perhaps within, the chorion. This view Guillemeau and Mauriceau had been led to take by reasoning; it has been verified post mortem by Duclos. But it may possibly not hold good of every case. As to the origin of this fluid he can offer no hypothesis. The practical conclusions which he submits are the following: He thinks metrorrhœa (a word proposed by Chassinant) a better name than hydrorrhœa, because it indicates that the phenomenon has nothing in common with the escape of the liquor amnii resulting from a premature rupture of the membranes. There are, perhaps, two forms of the disease—the one traumatic, the other catarrhal. It is very rare, and varies much in its clinical history. It is rarer the further the pregnancy is from term ; but it has been observed as early as the fifth week. As a rule it interferes neither with the course of the pregnancy nor the development of the fœtus ; but it may lead to abortion or to premature labor.

Œsophagism.—Dr. Eloy concludes (*Gaz. Hebd.*) an elaborate paper on Œsophagism in these terms: As a result of the consideration of all the cases which we have cited, we come to the conclusion that the most efficacious means for combating this affection, when existing independently of all functional disturbance—that is, œsophagism from a nervous cause, whether local or general, are the employment of catheterism (with or without dilatation) as a mechanical agent, the hypodermic injection of morphia as an analgesic, and the bromide of potassium (either by the mouth or as an enema) as a moderator of the reflex power. Moreover, this last agent will facilitate catheterism, by imparting a greater toleration of the mucous membrane to the contact of instruments."—*Medical Times and Gazette.*

The next annual meeting of the Illinois State Medical Society will be held in the city of Chicago, beginning on the 17th day of May next, and to continue during the following days till the business of the Society is accomplished. The committee of arrangements have secured the hall in the Methodist Church Block for the place of meeting, and hope that the attendance will be unusually large. All those expecting to read papers of any kind are requested to notify the chairman of the committee of arrangements, Dr. J. N. Hyde, of Chicago, at as early an hour as practicable. The profession in the city are requested to respond to the efforts of the committee toward making the forthcoming meeting as successful in a scientific and social point of view as is desired by all who are interested.

